# Clinical controversies in anticoagulation monitoring and antithrombin supplementation for ECMO

**DOI:** 10.1186/s13054-020-2726-9

**Published:** 2020-01-20

**Authors:** Meghan M. Chlebowski, Sirine Baltagi, Mel Carlson, Jerrold H. Levy, Philip C. Spinella

**Affiliations:** 1 Department of Pediatrics, Division of Pediatric Cardiology, Cardiovascular Intensive Care Unit, Cincinnati Children’s Hospital/University of Cincinnati College of Medicine, 3333 Burnet Ave, Cincinnati, OH 45229 USA; 2 Department of Pediatrics, Division of Critical Care Medicine, St. Joseph’s Children’s Hospital/University of Pittsburg School of Medicine, Tampa, FL USA; 3 LFB-USA, Framingham, MA USA; 4 Department of Anesthesiology, Critical Care, and Surgery, Duke University School of Medicine, Durham, NC USA; 5 Department of Pediatrics, Division of Pediatric Critical Care Medicine, Washington University School of Medicine, St. Louis, MO USA

**Keywords:** Anticoagulation, Antithrombin, Extracorporeal membrane oxygenation

## Abstract

During extracorporeal membrane oxygenation (ECMO), a delicate balance is required to titrate systemic anticoagulation to prevent thrombotic complications within the circuit and prevent bleeding in the patient. Despite focused efforts to achieve this balance, the frequency of both thrombotic and bleeding events remains high. Anticoagulation is complicated to manage in this population due to the complexities of the hemostatic system that are compounded by age-related developmental hemostatic changes, variable effects of the etiology of critical illness on hemostasis, and blood-circuit interaction. Lack of high-quality data to guide anticoagulation management in ECMO patients results in marked practice variability among centers. One aspect of anticoagulation therapy that is particularly challenging is the use of antithrombin (AT) supplementation for heparin resistance. This is especially controversial in the neonatal and pediatric population due to the baseline higher risk of bleeding in this cohort. The indication for AT supplementation is further compounded by the potential inaccuracy of the diagnosis of heparin resistance based on the standard laboratory parameters used to assess heparin effect. With concerns regarding the adverse impact of bleeding and thrombosis, clinicians and institutions are faced with making difficult, real-time decisions aimed at optimizing anticoagulation in this setting. In this clinically focused review, the authors discuss the complexities of anticoagulation monitoring and therapeutic intervention for patients on ECMO and examine the challenges surrounding AT supplementation given both the historical and current perspectives summarized in the literature on these topics.

## Background

During extracorporeal membrane oxygenation (ECMO), anticoagulation is required to prevent thrombotic complications, and unfractionated heparin (UFH) remains the predominant anticoagulation agent in this setting [1–3]. Heparin binds to antithrombin (AT) to potentiate its effect to inhibit thrombin 1000- to 2000-fold. The reduced ability of heparin to inhibit thrombin and fibrin formation is often termed “heparin resistance” and represents an alteration of heparin dose responses. Heparin resistance is recognized when there is a need for increasing doses of heparin to achieve the desired anticoagulation effect. Heparin resistance is a specific concern for patients on ECMO since AT activity is commonly decreased [2]. In addition, neonates have developmentally low AT levels, which may further contribute to the development of heparin resistance [4]. However, the minimal AT activity required for adequate heparin effect is unknown and validated, and age-appropriate thresholds for maintaining specific AT activity in ECMO patients do not exist. This has been difficult to establish because the relationship between AT activity and the resultant effect on reducing thrombin and fibrin formation with patient outcomes is dependent on multiple factors [5–7]. As such, AT supplementation for patients on ECMO in the setting of heparin resistance is controversial both in the pediatric and adult populations [8]. Table 1 demonstrates some of the challenges associated with achieving optimal anticoagulation in both neonatal and pediatric populations. These challenges are further compounded by the potential inaccuracy of the diagnosis of heparin resistance based on the use of single standard laboratory parameters used to assess heparin effect.

**Table 1:** Challenges to use of anticoagulation in neonates and children

◦ Developmental hemostasis ◦ Limited PK and pharmacodynamics data for anticoagulation agents ◦ Different epidemiology of thromboembolism and risks of anticoagulation therapy ◦ Fewer pediatric formulations of common anticoagulation agents ◦ Restricted diagnostic evaluation due to need of sedation for dx studies ◦ Irregular anticoagulation therapy monitoring due to difficult vascular access ◦ Inadequate validation of current diagnostic and treatment algorithms ◦ Lack of widespread experience and limited expertise ◦ Required collaborative approach with a multidisciplinary team ◦ Compliance concerns with high reliance on caregivers	

Saini A, Spinella PC: Management of anticoagulation and hemostasis for pediatric extracorporeal membrane oxygenation. Clin Lab Med 2014, 34(3):655–673

Adapted from Monagle P, Newall F, Campbell J. Anticoagulation in neonates and children: pitfalls and dilemmas. Blood Rev 2010; 24:151–62

## Bleeding and thrombosis during ECMO

Extracorporeal life support (ECLS) or ECMO use is expanding worldwide, with growth in both patient volume and total number of centers reporting to the Extracorporeal Life Support Organization (ELSO) [9]. According to the most recent report, survival to hospital discharge rates for adults requiring ECMO are 57% for patients with respiratory illnesses and 42% for cardiac disease [9]. Pediatric ECMO cases have also increased 24% from 2009 and 2015 with a concurrent 55% growth in pediatric ECLS centers [10]. In 2015, survival rates were approximately 61% for neonatal respiratory, pediatric respiratory, and pediatric cardiac ECMO cases, compared to approximately 42% in neonatal cardiac as well as neonatal and pediatric extracorporeal cardiopulmonary resuscitation [10].

**Fig. 1:**
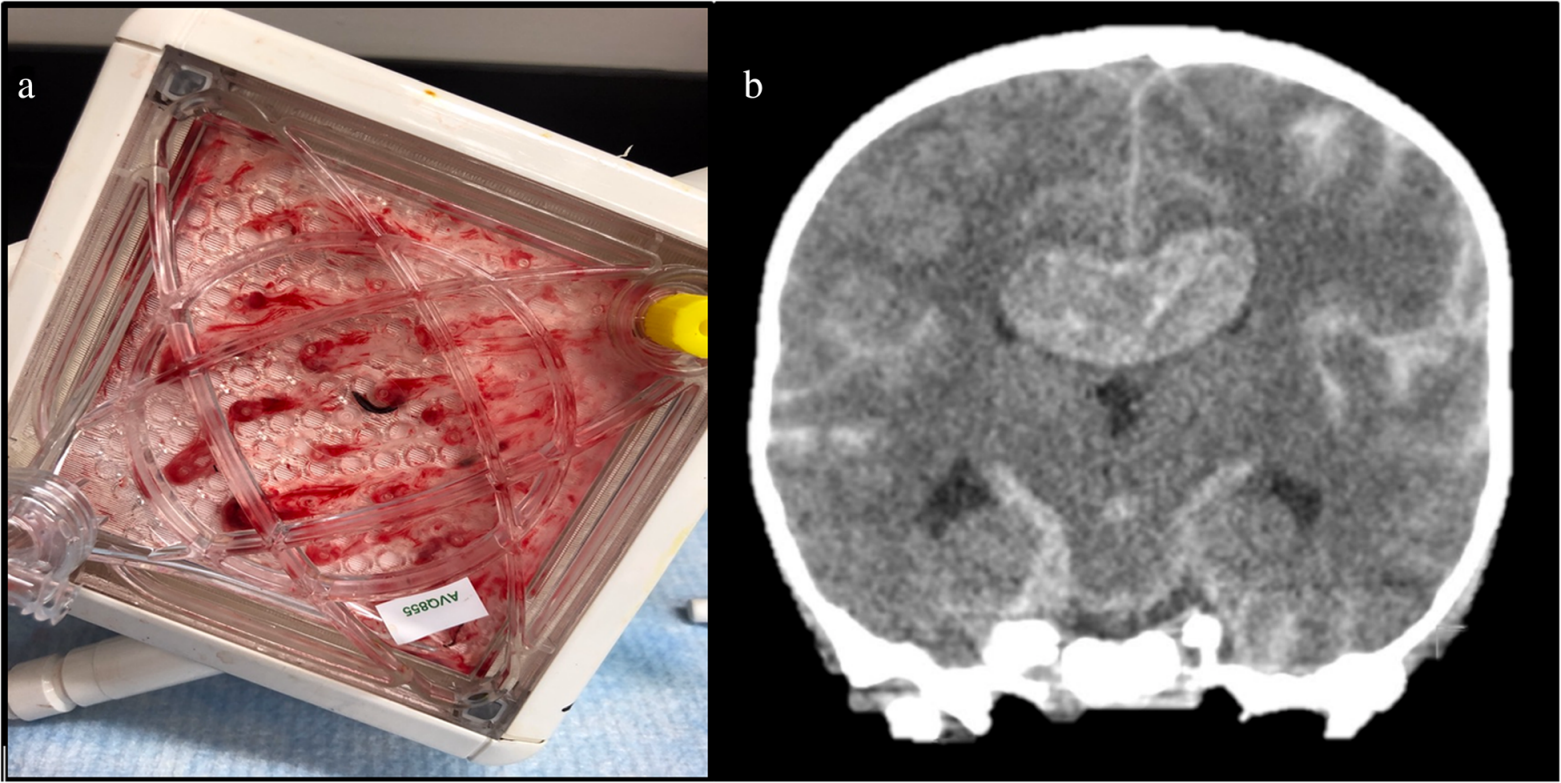
Balance between thrombotic and bleeding complications on ECMO. Panel (**a**) demonstrates significant thrombus burden in the oxygenator. Panel (**b**) demonstrates a bleeding complication of a large intraventricular hemorrhage

As seen in Fig. 1, bleeding and thrombotic complications during ECMO are common and have a significant impact on patient outcomes [9–11]. In a 2017 study of pediatric ECMO cases involving 8 centers and 514 patients under 19 years, bleeding occurred in 70% of cases, including 16% involving intracranial hemorrhage, which was independently associated with a higher risk of mortality. Thrombotic complications occurred in 13% of pediatric patients with 31% of cases requiring circuit component change [12]. In addition, in an autopsy series of 29 children with ECLS, thrombosis and hemorrhage were common, with one or both observed in 86% of patients [11]. Similar data exists for adults showing that coagulation disorders for adult patients on ECMO have been reported to be as high as 33% [13]. However, as a postmortem analysis of 78 consecutive adult ECMO deaths showed, there is a high rate of clinically unrecognized venous thromboembolism (VTE) and systemic thromboembolic events in up to 32% of cases suggesting that the incidence of thrombotic events in ECMO may be underreported when relying on clinical evaluation alone [14].

## Methods for anticoagulation monitoring

In the absence of preexisting coagulopathy, hemostatic dysfunction during ECMO occurs as a consequence of sheer stress and exposure of blood to the non-biologic surfaces of the ECMO circuit [15, 16]. Mechanical forces provoke activation of platelets and coagulation factors, fibrinogen deposition, adherence to device surfaces, and thrombin generation. High sheer stress also changes the configuration of von Willebrand factor, cleaves high molecular weight fragments, and increases consumption to increase bleeding [17]. In addition, the underlying critical illness of the patient, including the presence or absence of cardiogenic shock with shock liver, sepsis-induced coagulopathy, and/or disseminated intravascular coagulopathy, can directly or indirectly through immune and endothelial activation pathways can alter hemostasis.

Numerous variables must be considered for optimal ECMO anticoagulation. These include various patient factors including patient age, underlying illness, duration of ECMO, heparin dose, target antithrombin activity, and risk of thrombotic or bleeding events. In addition, the selection and schedule of diagnostic tests including platelet count, antithrombin (AT), activated clotting time (ACT), activated partial thromboplastin time (aPTT), anti-factor Xa (anti-Xa), prothrombin time and international normalized ratio (PT/INR), thromboelastography (TEG®), or rotational thromboelastometry (ROTEM) must be carefully considered [18]. Achieving an appropriate balance between preventing thrombosis and the risk of bleeding is further complicated by the fact that standard diagnostic tests are only partial functional measures of hemostasis. In other words, while coagulation tests are used to guide anticoagulation, they do not always accurately predict clinically relevant hemostasis-related outcomes including risk for thrombosis or bleeding. Currently available functional hemostasis monitoring assays are imprecise and have not been established to accurately reflect thrombin formation or predict the risk for excessive bleeding in any patient population or during ECMO [19, 20]. As a result, anticoagulation monitoring and management remain significant challenges for the physician managing a patient on ECMO [21]. In order to better understand the strengths and limitations of each diagnostic test and its role in anticoagulation monitoring on ECMO, the most widely utilized whole blood and plasma-based tests to assess hemostasis for patients on ECMO will be reviewed.

### Activated clotting time

The ACT is a whole blood test used to measure the anticoagulant effect of heparin [2, 22]. The ACT is performed by adding whole blood to a tube containing a surface activator, either kaolin or diatomaceous earth, which stimulates the contact activation pathway. The ACT is a clot-based assay that measures either the mobility of a magnet during clot formation or the change in velocity of movement through blood as it clots. The time for initial fibrin formation within the tube is measured in seconds. Multiple factors can prolong the ACT independent of UFH dose, including hemodilution, platelet function and number, hypothermia, hypofibrinogenemia, and coagulation factor deficiencies [23, 24]. In contrast to the 400–800-s target for cardiopulmonary bypass, an ACT range of 180–200 s has been suggested for ECMO [25]. However, different ACT platforms and their relationship to measured heparin levels and aPTT are inconsistent, especially in the lower ACT target ranges for ECMO [26, 27]. As a result, ACTs have a poor correlation with heparin concentrations within the dose range typically used for ECMO [28–31]. In addition, Bembea et al. demonstrated poor correlation with ACT and anti-Xa activity (*r* = 0.02) in 34 ECLS pediatric patients despite a good correlation between ACT and heparin infusion dose within each patient (*r* = 0.77) [32]. While there was no association with discordant ACT to anti-factor Xa values in patients who experienced thrombotic complications requiring a circuit change, there was a significant discordance between ACT and anti-factor Xa values in patients who had a hemorrhagic complication compared to those who did not (ACT < 180 s and concomitant anti-factor Xa > 0.7 IU/mL) [32]. This suggests that the anti-Xa assay is a better correlate of heparin levels when compared to aPTT or ACT in pediatric ECMO [33]. Similar results have also been demonstrated in adult ECLS patients. In a retrospective analysis of ACT measurements taken during ECMO procedures, there was a poor correlation (*r* = 0.11–0.14) between ACT values and heparin dosing [34]. The authors also reported no association between ACT and aPTT when paired samples were divided into subtherapeutic, therapeutic, and supratherapeutic groups, and concluded that ACT is an unreliable tool to monitor UFH during ECLS in adults.

### Activated partial thromboplastin time

The aPTT test is a plasma-based assay of clot formation used to monitor UFH. Therapeutic ranges for ECMO are 60 to 80 s in the setting of standard bleeding risk versus targets of 40 to 60 s in patients at an increased bleeding risk [18, 35, 36]. The PTT test is performed by mixing citrated plasma with silica, a synthetic phospholipid (ellagic acid), and calcium to initiate clot formation. Different analytic methods exist based on either optical or mechanical clot detection. In the optical method, clot formation is measured by a change in optical density, whereas mechanical clot detection monitors the movement, or oscillations, of a steel ball within the test solution. As fibrin is formed, the rate of oscillation slows and it is at this time that the PTT is measured. Similar to the ACT, an aPTT test evaluates contact activation in the intrinsic pathway and is affected by fibrinogen and factor VIII levels [37]. A standard laboratory-based aPTT uses plasma, but whole blood point of care tests are available. There are more than 300 laboratory methods used to monitor aPTT with different results obtained depending on the method utilized [38]. For example, at a plasma heparin concentration of 0.3 IU/mL measured by factor Xa inhibition, aPTT results can range from 48 to 108 s depending on the reagent used [39]. As a result of this wide variation, the PTT target range used at one ECMO center should not be translated to other centers without confirming the type of assay used for aPTT.

Currently, 94% (109/116) of ELSO-reporting centers check aPTT daily at varying frequencies [2]. Several investigators have examined the correlation between aPTT, ACT, and anti-Xa activity. In neonates, Khaja et al. demonstrated an improved correlation of aPTT with anti-Xa results when compared with ACT [31]. Similar results were observed in the adult ECMO population. A retrospective study of 46 patients demonstrated little or no correlation between ACT and heparin dose, a moderate correlation between aPTT and heparin dose, and a weak correlation between ACT and aPTT [34]. However, another study demonstrated that despite improved correlation of aPTT to anti-Xa activity, 44.2% of measurements were discordant [33]. This argues against the use of aPTT alone in assessing the adequacy of anticoagulation or as an accurate measure of heparin effect in the context of diagnosing heparin resistance.

### Anti-factor Xa

The anti-Xa concentration directly measures heparin inhibition of factor Xa and is increasingly used to measure heparin effect, especially in pediatric patients. Target values during ECMO range from 0.3 to 0.7 IU/mL [31, 40]. Anti-Xa assay kits can be affected by hyperbilirubinemia and high plasma free hemoglobin which can occur in ECLS patients and falsely lower anti-Xa activity. For example, ECMO samples with plasma free hemoglobin samples of 50 mg/dL or greater had significantly lower anti-Xa activity compared with normal: 0.33 (0.25–0.42) versus 0.4 (0.31–0.48) IU/mL [31, 40, 41].

As previously noted, the anti-Xa assay correlates better with heparin concentration than with ACT or aPTT [33, 42]. In a single-center study of 12 neonatal ECMO patients, there was a strong correlation between anti-Xa assays and heparin dose (*r* = 0.75; *p* < 0.0001), while the ACT did not correlate with either anti-Xa assays or heparin dose [30]. An observational cohort study of 34 pediatric ECMO patients further confirmed these observations demonstrating a moderate correlation with heparin concentrations when measured simultaneously (*r* = 0.33) while also showing poor correlation with ACT and aPTT (*r* = 0.02 and 0.17, respectively) [32].

Criticism of using anti-Xa values in isolation to titrate heparin for anticoagulation is that while it is a direct measure of heparin effect, it does not represent the overall hemostatic state of the patient. Anti-Xa values represent the amount of inhibition, not the amount of thrombin and fibrin that is able to be generated in the patient. One view is that the amount of inhibition needs to be put in context with the amount of thrombin and fibrin being produced in a patient. For example, a patient who before heparin therapy is highly prothrombotic may still be prothrombotic with what is considered to be a therapeutic effect of heparin based on anti-Xa levels. Anticoagulation titration may be more effective when it is titrated according to the net amount of thrombin and fibrin formation instead of how much Xa is being inhibited.

### Viscoelastic tests

Thromboelastography (TEG®) and rotational thromboelastometry (ROTEM) are viscoelastic tests of hemostasis in whole blood that have been used to monitor anticoagulation with ECMO [43]. TEG®/ROTEM parameters inform time to initial fibrin formation, crosslinking of fibrin, clot firmness, platelet function, and fibrinolysis. Paired TEG®/ROTEM samples with and without the addition of heparinase allow for the underlying assessment of hemostasis in the presence of UFH. As a result, UFH responsiveness can be evaluated with TEG®/ROTEM by examining the difference in R or clotting time (CT) between tests with and without heparinase, which may be beneficial when there is concern for heparin resistance.

In a series of 27 pediatric ECLS patients, TEG® measurements were performed alongside ACT and aPTT [44]. In 171 paired results, aPTT correlated with all TEG® parameters (R time, K time, and α angle), but given that they both measure time to initial fibrin formation, the strongest correlation was between aPTT and TEG® R time (*r* = 0.31). In contrast, ACT correlated weakly with all TEG® parameters. Similar results have been published comparing ROTEM with conventional coagulation tests [45]. To date, there are no large multicenter trials comparing viscoelastic tests with conventional coagulation measures and their ability to guide anticoagulation therapy. One small pilot RCT was performed indicating that it is feasible to conduct such a trial [46].

TEG has been reported to be useful in identifying various hypercoagulable conditions including those associated with major surgery and malignancy [47–51]. Thromboelastometry analyses are increasingly included in the evaluation of global clotting function and monitoring of hemostatic treatment in various clinical situations including liver transplantation, cardiac surgery, obstetrics, trauma, and hemophilia [52–63]. It is also commonly used to guide transfusion management [64, 65]. In regard to patients supported on ECMO, multiple studies have evaluated the safety and feasibility of a TEG-driven strategy to titrate heparin versus the “conventional” approach based on aPTT monitoring with a trend toward improvement in adverse outcomes. For example, a recent multicenter, randomized, controlled trial was performed involving adult patients with acute respiratory failure treated with venovenous ECMO who were randomized to manage heparin anticoagulation using a either a TEG-based protocol (target 16–24 min of the R parameter, TEG group) or a standard of care aPTT-based protocol (target 1.5–2 of aPTT ratio, aPTT group). While underpowered to detect statistically significant differences between the groups (*N* = 42), patients in the aPTT group tended to bleed more compared to the TEG group (15 vs. 10, *p* = 0.21). In addition, heparin dosing was lower in the TEG group compared to the aPTT group (12 IU/kg/h vs. 16 IU/kg/h, respectively, *p* = 0.03) with no increase in thrombotic complications. While a larger trial is needed, the results are encouraging that a TEG-driven protocol is both safe and feasible in adult patients requiring V-V ECMO [46]. In a recent pediatric study, a retrospective chart review of patients requiring venovenous (VV) and venoarterial (VA) ECMO was performed within a single-center, tertiary care children’s hospital. The study evaluated optimal values for citrated kaolin TEG R time and anti-Xa activity that would minimize both bleeding and thrombotic complications in pediatric and neonatal patients. The study concluded that an anti-Xa activity greater than .25 IU/mL (sensitivity 81%, specificity 67%, PPV 81%, and NPV 58%) and a TEG R time greater than 17.85 min (sensitivity 84%, specificity 68%, PPV 82%, and NPV 59%) may minimize the risk of thrombosis in pediatric and neonatal ECMO patients. An optimal target to minimize the risk of bleeding events was unable to be identified in this study [66].

### Antithrombin

Antithrombin (AT), a plasma alpha-2 glycoprotein, plays a central role in the physiologic plasma anticoagulation system. Normal plasma concentration is 15–20 mg/dL and circulates at 2.4 μM concentrations [67]. Its action includes the irreversible inhibition of multiple clotting factors including thrombin (factor IIa), factor Xa, and to a lesser extent factors IXa, XIa, XII, tissue plasminogen activator, plasmin, and kallikrein. In its natural form, AT has a low level of anticoagulant activity. However, in the presence of heparin, anticoagulant activity is enhanced 1000–2000-fold [68]. Independent of its anticoagulant properties, AT also has important anti-inflammatory attributes when binding to the endothelium via heparin-like glycosaminoglycans resulting in prostacyclin release [69, 70]. Prostacyclin inhibits leukocyte activation by inhibiting tumor necrosis factor-alpha production, and limits neutrophil activation and adhesion to endothelial cells [71].

In general, young children have decreased physiologic concentrations of anticoagulant proteins, including antithrombin. This is most pronounced in children less than 1 year of age as compared with older children and adolescents. Antithrombin levels reach adult levels by the age of 7–12 months; however, reference ranges for antithrombin levels even in healthy children differ significantly by age and can vary depending on the reagents and analyzers utilized [72].

In addition, children have reduced activity of procoagulant proteins (factors II, VII, IX, XI, and XII) and inhibitors of coagulation (protein C, S, antithrombin) compared to adults while fibrinolytic activity is decreased, particularly in neonates, due to the slow activation kinetics of tissue plasminogen activator (t-PA) on neonatal plasminogen coupled with normal to elevated levels of plasminogen activator inhibitor (PAI) at birth [73, 74]. As a result, because the balance of pro- and anticoagulant proteins is different in adults, children, and infants, it is difficult to ascertain the effect of AT activity on overall hemostatic function in isolation. This further complicates both the interpretation and decision-making surrounding supplementation in patients on ECMO.

When AT is supplemented for patients with hereditary AT deficiency, the hemostatic system is otherwise normal. As a result, the hemostatic effect of AT can be more easily predicted. This is in contrast to the patients on ECMO where both their underlying illness and the blood-circuit interaction can alter the underlying hemostatic balance. This makes the effect of AT supplementation less predictable. In pediatric and neonatal patients, hemodilution can further decrease AT levels due to the volume of blood in the ECMO circuit relative to patient blood volume [75]. As a result, timely assessment of antithrombin activity with other functional measures of hemostasis should be considered, and perhaps, a systems biology-based approach is needed for interpreting functional measures of hemostasis in critically ill patients on ECMO.

## Limitations of hemostasis monitoring and their interpretation

The goal of heparin anticoagulation is to reduce thrombotic events in the ECMO circuit and patient by reducing thrombin and fibrin formation. The goal of heparin anticoagulation is not to achieve a specific heparin effect in isolation of the patients’ overall hemostatic capacity or clinical state. As such, the heparin effect must be interpreted in the context of the amount of thrombin and fibrin that is being produced before heparinization is initiated. In addition, the overall hemostatic capacity of the patient based on measures of fibrinogen and platelet function should also be considered when interpreting the effect of heparin on a patient.

As this review suggests, assays such as anti-Xa provide an accurate result for heparin effect, but do not provide information on the overall functional effect of thrombin and fibrin formation. ACT, aPTT, and viscoelastic values do provide more global measures of hemostasis but are limited by their lack of incorporating high sheer stress and biological surfaces, such as collagen or endothelial cells, that are essential for hemostasis. It is the overall net effect of thrombin and fibrin formation reduction with heparinization that is clinically important, not the amount of inhibition of factor Xa or direct heparin effect.

In an era of goal-directed therapy, the goal of heparinization is to reduce thrombin activity and fibrin formation to decrease thrombotic events without causing bleeding. To accomplish this, the titration of anticoagulation therapies should include global measures of thrombin and fibrin formation with viscoelastic assays, not only the amount of inhibition of factor Xa or the aPTT in isolation. In addition, caution should be used when interpreting analyses that assess a correlation between hemostatic assays, including anti-Xa, as there is no data to suggest that maintaining an anti-Xa concentration within a particular range is associated with improved outcomes.

Similar issues exist when interpreting AT activity and heparin resistance, particularly in isolation without the context of the overall hemostatic state of the patient. For example, the isolated use of anti-Xa values to determine heparin resistance may result in AT replacement in situations where there is already adequate suppression of thrombin/fibrin formation which can increase the risk of bleeding. Conversely, the measurement of AT activity in isolation can also lead to inadequate AT supplementation if the patients are in a hypercoagulable state with high thrombin/fibrin formation despite an anti-Xa that is “in range” and considered to be therapeutic.

The following sections discuss AT replacement according to AT activity since it is the current standard practice. The future of heparin anticoagulation management and AT supplementation may change with the advent of improved hemostatic monitoring that can more accurately provide a comprehensive assessment of the patient’s overall hemostatic function that reflects an accurate assessment of thrombin/fibrin formation.

## Antithrombin replacement in ECMO

### Available products

In the United States (US), antithrombin is available in either human plasma-derived form (Thrombate III®; Grifols, Research Triangle Park, NC) [76] or recombinant form (ATryn®; LFB, rEVO Biologics, Framingham, MA) [77]. Thrombate III® is prepared from pooled donor plasma with a multistep purification process whereas recombinant antithrombin (ATryn®) is prepared from the milk of genetically engineered goats. Thrombate III® is approved for the treatment and prevention of thromboembolism in patients with hereditary antithrombin deficiency, whereas ATryn® is indicated for the prevention of perioperative and peripartum thromboembolic events in hereditary antithrombin-deficient patients. Acquired antithrombin deficiency is more common than congenital antithrombin deficiency requiring off-label use of AT concentrate. A review of the US Pediatric Health Information System database noted that 97% of children who were treated with AT received it off-label, with neonates as the largest population (46%). The most common diagnosis associated with off-label use was congenital heart/lung problems (36%), and the most common procedure was ECMO (44%) [78].

The elimination half-life of plasma-derived AT concentrate (Thrombate III®) is 2.5–3.8 days vs. 12–18 h for recombinant human AT concentrate (ATryn®). Thrombate III® is administered by intravenous bolus infusion, whereas ATryn® is administered as an initial intravenous infusion loading dose followed by continuous infusion. Table 2 profiles the dosing formulae for both products. Once reconstituted, plasma-derived AT concentrate must be administered within 3 h vs. 8–12 h for recombinant AT concentrate. As with any factor concentrate, Thrombate III® has the potential to transmit infectious agents particularly variant Creutzfeldt-Jakob disease (vCJD) and theoretically Creutzfeldt-Jakob (CJD) disease. However, no cases have been reported [77, 79].

**Table 2:** Dosing for antithrombin

	Surgical dosing	Obstetrical dosing
Plasma-derived AT concentrate [56]		
Initial loading dose (IU)	([desired % AT activity − baseline % AT activity] × body weight in kilograms) ÷ 1.4 Infuse IV over 10–20 min	Same formula
Maintenance dose (IU)	60% of the initial loading dose, given every 24 h for 2–8 days	
Dose adjustments	Adjust maintenance dose and/or interval to maintain AT activity levels of 80–120% Measure AT activity levels 20 min postinfusion of initial dose, every 12 h, and before each infusion	
Recombinant AT concentrate [57]		
Initial loading dose (IU)	([100 − baseline % AT activity] ÷ 2.3) × body weight (kg) Administer loading dose as a 15-min intravenous infusion and immediately follow it by a continuous infusion of the maintenance dose	([100 − baseline % AT activity] ÷ 1.3) × body weight (kg) Administer initial dose as a 15-min intravenous infusion and immediately follow it by a continuous infusion of the maintenance dose
Maintenance dose (IU/h)	([100 − baseline % AT activity] ÷ 10.2) × body weight (kg) given per hour	([100 − baseline % AT activity] ÷ 5.4) × body weight (kg) given per hour
Dose adjustments	Adjust based on the % AT activity level 2 h after initiation of treatment For AT activity level < 80%, increase dose by 30% and recheck 2 h after each dose adjustment For AT activity level 80–120%, do not adjust and recheck 2–6 h after initiation of treatment or dose adjustment For AT activity level > 120%, decrease dose by 30% and recheck 2 h after each dose adjustment	

*AT* antithrombin, *IU* international unit, *%* percentage, *IV* intravenous, *kg* kilogram, *h* hour

### Heparin resistance

Heparin resistance occurs when there is a need for increasing doses of heparin to achieve the desired anticoagulation effect based on anti-Xa, PTT, or ACT. Conventional treatment of heparin resistance includes administering increasingly higher doses of heparin to bind all available AT, supplementing with exogenous AT concentrate, or transfusing fresh frozen plasma (FFP) as a source of AT [24]. Additional heparin may prove ineffective if AT is severely deficient, and ceiling effects have been reported [80]. Although FFP can be used as a source of antithrombin, it requires cross-matching and carries the potential risk of transfusion-transmitted infectious disease. In addition, FFP contains only 1 U/mL of AT, so doses of approximately 20 mL/kg could be required to restore AT to normal levels [81]. This can lead to transfusion-associated circulatory overload (TACO) or transfusion-related acute lung injury (TRALI) [82].

### Review of data surrounding antithrombin replacement strategies and controversies

Traditionally, AT dosing is based on patient weight and desired AT activity level expressed as a percent between 80 and 120% (see formula in Table 2) [76, 77]. However, the optimal target threshold for antithrombin during ECMO has not been clearly defined or validated, particularly when patients are fully heparinized. In a recent international survey of anticoagulation management during ECLS, respondents reported highly variable target AT ranges from as low as 30% to as high as 120%. Furthermore, antithrombin level monitoring is also highly variable with levels checked routinely in 60 centers, occasionally in 26, and never in 21 centers [2].

In patients with congenital AT deficiency, continuous infusions have been reported to stabilize AT blood levels and reduce bleeding complications when compared to bolus dosing [83]. However, no data exists indicating if bolus or dosing by continuous infusion affects clinical outcomes for patients on ECMO. A recent pediatric retrospective case-controlled study examined continuous infusion of antithrombin (ATryn®) compared to intermittent bolus doses (Thrombate III®) on ECMO. This study suggested that AT administered by continuous infusion increased the time that ACT stayed within goal range, lowered the heparin dose, did not increase hemostatic complications, demonstrated a trend toward fewer heparin dose adjustments, and lowered blood product usage. However, this study was limited by small sample size (*n* = 14) and historical case control design [83].

A review of the literature discussing AT supplementation based on an AT target activity level is summarized in Additional file 1. The table shows variability in AT levels and in the patient population studied. In most studies, AT was given at the discretion of the physician with no explanation of the indication. In others, AT administration was protocol driven. There was also high variability in the dose of antithrombin administered and the outcomes studied. In addition, it was not clear if the indication for AT replacement was based on a single AT activity value or a panel of hemostasis results. This makes generalizing data from the current literature on AT supplementation difficult. Similarly, the relationship between AT activity and global hemostatic function has not been examined closely. An investigation into interactions between AT and other pro- and anticoagulant factors, including their effect on thrombin formation and platelet activity, is needed to improve the interpretation of AT supplementation and activity. Prospective multicenter studies are needed to identify and evaluate clinical and diagnostic indications for the administration of AT, including threshold, dose, duration, and outcomes.

## Outcomes in ECMO relating to AT supplementation

Overall, there are limited studies evaluating anticoagulation management, antithrombin replacement, and outcomes for patients on ECMO. In a retrospective study of 22 pediatric ECMO patients, O’Meara and colleagues used an anti-Xa guided protocol to manage anticoagulation compared to an historical ACT-based control group. All patients received AT using the following criteria: (a) when AT is < 50% or (b) when AT is less than 100% and heparin requirements exceed 45 U/kg/h. The control group using ACT fell outside of the target goal 22% of the time compared to 9% in the anti-Xa group. Bleeding complications occurred in 27% of the anti-Xa cases vs. 50% in the ACT group. The authors concluded that consistent management of anti-Xa levels within the therapeutic range (0.4–0.8 IU/mL) might decrease the incidence of thrombus formation [84].

These observations are consistent with research conducted by Irby and colleagues who retrospectively analyzed 62 pediatric ECMO patients using daily anti-Xa measurements. Two groups were identified: a group requiring no circuit change (mean anti-Xa concentration 0.20 IU/mL) and a group requiring circuit change due to thrombus formation (mean anti-Xa concentration 0.13 IU/mL). The study demonstrated that each 0.01 IU/mL decrease in anti-Xa activity increased the odds for a circuit or oxygenator change by 5%, suggesting that the low anti-Xa group displayed 41% increased odds for risk of circuit change [85].

In 2011, a tertiary care academic children’s hospital altered their anticoagulation protocol to include anti-Xa, thromboelastography, and AT. Goal anti-Xa levels were 0.3 to 0.7 units/mL, and antithrombin was replaced if the AT level was low for age and heparin requirements were > 60 U/kg/h. Following implementation of the new protocol, cannula and surgical site bleeding decreased from 22 to 12%, and 38 to 25%, respectively, with decreased transfusion of red cells, fresh frozen plasma, platelets, and cryoprecipitate. Median membrane circuit life increased from 3.6 to 4.3 days, and survival to hospital discharge increased from 43 to 55% (*p* = 0.06) [86].

In a retrospective cohort study by Kessel et al. [87], 18 pediatric patients received ECMO support between January 2004 and March 2013. For the duration of each ECMO circuit use, a new, multifactorial approach using ACT, aPTT, PT/international normalized ratio (INR), anti-Xa level, and AT activity was used to titrate the heparin infusion dose. Nine patients were age and diagnosis matched with nine patients based on ACT only. The modified monitoring regimen slightly increased the amount of time that anticoagulation parameters were within range compared to the ACT-only group.

Finally, a recent retrospective study analyzed the influence of antithrombin levels on aPTT, ACT, INR, bleeding, thrombus formation, kaolin + heparinase TEG alpha angle, kaolin TEG reaction time, heparin dose rate (HDR), anti-Xa, bivalirudin dose rate, argatroban dose rate, interventions, and transfusions. Thirty-five infant-pediatric patients underwent ECLS between January 2013 and January 2016 who remained on ECLS for at least 5 days. No significant correlation between optimal aPTT and HDR at various AT levels was found. However, receiver operating characteristic (ROC) analysis suggested that to maintain an aPTT above 60 s, an AT threshold of 42% or higher was observed when the HDR was > 12 U/kg/h. ROC analysis also determined that no thrombus was associated with an aPTT > 64 s and decreased bleeding was associated with a kaolin TEG reaction time below 30 min [7].

## Limitations

Many of the studies included in this review were retrospective, single-center studies with different endpoints, different study designs, and different sample sizes. In order to provide a comprehensive review of the clinical controversies, the authors included relevant neonatal, pediatric, and adult studies. Since these patient populations have different developmental hemostatic differences and disease processes, inferring universal conclusions to the general population is difficult and one must take into account such differences when reviewing and applying this data. In addition, each study utilized different therapeutic targets, different dosages, and different formulations making it difficult for physicians to infer conclusions in regard to dosing of UFH, timing and therapeutic targets for AT supplementation, and desired outcomes. Ultimately, if funding is available, a meta-analysis of both pediatric and adult literature surrounding anticoagulation management and AT supplementation on ECMO might prove beneficial.

## Summary and recommendations

Current ELSO guidelines for anticoagulation during ECMO recommend an initial heparin infusion rate of 7.5–20.0 units/kg/h [88]. With this large range and little guidance regarding which laboratory tests to monitor, many institutions have turned to literature and experience to develop their own heparin protocol for ECMO. A survey study found that anticoagulation management policies vary greatly by center [2], and another study found that the use of a standardized anticoagulation protocol is associated with a decrease in hemorrhagic complications [86]. In addition, the guidelines suggest supplementing AT during ECMO only when its deficiency coexists with heparin resistance. As this review suggests, this approach may not be sufficient. The actual therapeutic AT target needed to achieve adequate anticoagulation is unknown, and correcting an AT level to an arbitrary target endpoint may result in an adverse outcome such as thrombosis or bleeding. In addition, this review has shown that the use of one laboratory test to monitor anticoagulation effect and determine therapeutic interventions is insufficient. Instead, it may be more efficient for clinicians to examine the hemostatic system holistically with multiple laboratory tests that are interpreted in the context of the patient and ECMO circuit conditions. Ultimately, increased knowledge of AT use patterns and outcomes associated with its supplementation will help inform future trials to determine its efficacy and safety. In addition, the authors recommend the development of multicenter trials that examine outcomes according to either AT activity-based indications or overall reduced effect of heparin on thrombin and fibrin formation. Trials designed should include both age- and weight-based criteria to follow for both AT dosing and threshold recommendations. Furthermore, we would recommend population pharmacokinetic and pharmacodynamic modeling, as well as prospective trials, to delineate the superior means of adjusting heparin therapy and AT supplementation to prevent adverse clinical outcomes. There is currently an ongoing pilot prospective randomized controlled, single-blinded, multicenter study that is evaluating the efficacy of a protocol of AT supplementation in decreasing heparin dose and improving anticoagulation adequacy in adult patients supported on ECMO for respiratory failure [89]. Such studies should also be performed in both children and neonates.

Overall, our current recommendations are to use multiple laboratory tests including but not limited to anti-Xa, TEG®/ROTEM, PTT, and AT levels. At our center, we have developed an algorithm for managing anticoagulation that utilizes multiple laboratory tests along with a specific anticoagulation team that oversees the anticoagulation management of these patients and monitors for bleeding and thrombotic outcomes. Of all the aspects of clinical care in ECMO, anticoagulation is the least understood due to the myriad of complexities of coagulation physiology that are compounded by age-related developmental hemostatic changes, variabilities in critical illness, and changes within the blood-circuit interaction. As stated above, more studies are needed to develop more algorithms that would target different age groups including neonates, pediatric patients, and adults.

## Conclusions

Despite previously published ELSO anticoagulation guidelines from 2014, there remains no standardized method to achieve and monitor anticoagulation during ECMO [88]. Significant practice variation exists across centers concerning anticoagulation management as there is no evidence-based consensus regarding which tests, test thresholds, and interventions can optimize thrombotic and bleeding complications and outcomes [2].

Certain observations are noteworthy. First, despite its frequent use for ECMO anticoagulation, current research suggests that using ACT alone to guide anticoagulation may not be optimal. As a result, determining what additional tests should be evaluated and in what sequence is important to determine. In addition, our review of the literature suggests that monitoring antithrombin levels is an important component of any anticoagulation management protocol. Despite the heterogeneity of coagulation monitoring and interventions in ECMO centers, the implementation of evidence-based protocols for anticoagulation and transfusion is needed.

We believe that optimal anticoagulation, including the indications for antithrombin supplementation, relies on a comprehensive and standardized evaluation of multiple measures of hemostasis including aPTT, ant-Xa, TEG®, AT activity, platelet count, and fibrinogen concentration. Anticoagulation should be titrated based on the overall hemostatic state of the patient as evidenced by laboratory evaluation and should be put in context with the clinical hemostatic state of the patient and his or her unique risk of bleeding or thrombotic complications. Development of more accurate measures of hemostasis that can incorporate high sheer stress and biologic surfaces such as microfluidic models is needed to more accurately assess the hemostatic potential of patients on ECMO to allow for more precise titration of anticoagulation and antithrombin levels when indicated [90, 91].

## Supplementary information

**Additional file 1.** Summary of Antithrombin Studies in ECMO Patients. A review of the literature discussing AT supplementation based on an AT target activity level.

## Data Availability

Not applicable

## Abbreviations

ACT: Activated clotting time
Anti-Xa: Anti-factor Xa
aPTT: Activated partial thromboplastin time
AT: Antithrombin
CJD: Creutzfeldt-Jakob disease
CT: Clotting time
ECLS: Extracorporeal life support
ECMO: Extracorporeal membrane oxygenation
ELSO: Extracorporeal Life Support Organization
FFP: Fresh frozen plasma
HDR: Heparin dose rate
INR: International normalized ratio
IU: International unit
IV: Intravenous
KTEG R-time: Kaolin TEG reaction time
PAI: Plasminogen activator inhibitor
pRBCs: Packed red blood cells
PT: Prothrombin time
ROC: Receiver operating characteristic
ROTEM: Rotational thromboelastometry
TACO: Transfusion-associated circulatory overload
TEG®: Thromboelastography
t-PA: Tissue plasminogen activator
TRALI: Transfusion-related acute lung injury
U: Units
UFH: Unfractionated heparin
US: United States
VA: Venoarterial
vCJD: Variant Creutzfeldt-Jakob disease
VTE: Venous thromboembolism
VV: Venovenous
