# Supplementary material for: Clinical controversies in anticoagulation monitoring and antithrombin supplementation for ECMO
Source: Crit Care. 2020 Jan 20;24:19. doi: 10.1186/s13054-020-2726-9 (PMC6971875; doi:10.1186/s13054-020-2726-9)
Supplement: Supplementary file 1 — Additional file 1. Summary of Antithrombin Studies in ECMO Patients. A review of the literature discussing AT supplementation based on an AT target activity level. [file 13054_2020_2726_MOESM1_ESM.docx]

**Additional file 1. Summary of Antithrombin Studies in ECMO Patients**

| Author | Study | Population | N | AT Target | AT Mode of Delivery | Outcome |
| --- | --- | --- | --- | --- | --- | --- |
| Colman 2019 | Retrospective chart review stratified cohorts by study interval: pre-protocol (January 2016–March 2017) or post-protocol (March 2017–December 2017) | Adult ECMO  VA 80% of patients | pre-protocol = 72  post-protocol = 51 | AT > 50% with no evidence of therapeutic heparin effect | - Not specified | - 29 patients received AT vs. 94 patients who did not receive AT - No statistically significant difference in average heparin dosage over 48 hours, aPTT time in therapeutic range, major bleeding, or minor bleeding - The rate of thrombosis was higher in patients who received AT than in those who did not receive ATIII (48.2% *vs.* 15.9%; P<0.01). - No significant difference between these 2 groups in time to therapeutic anticoagulation (14 *vs.* 18 h; P=0.08) or in the percentage of patients who never reached a therapeutic heparin level in 48 hours (14% *vs.* 12%; P=0.76). |
| Morisette 2019 | Retrospective Observational | Adults ECMO | 14 | AT was supplemented when anti-Xa levels were subtherapeutic with unfractionated heparin infusion rates of 15-20 units/kg/h and measured AT <50%. |  | - The median percentage of time therapeutic anti-Xa levels were maintained was 0% (0-43%) and 40% (9-84%) in the pre-AT and post-AT groups, respectively (p = 0.13). - No difference was observed in the number of patients attaining a single therapeutic anti-Xa level (pre-AT = 6, post-AT = 13; p = 0.37) or unfractionated heparin infusion rate (pre-AT = 7.35 (1.95-10.71) units/kg/h, post-AT = 6.81 (3.45-12.58) units/kg/h; p = 0.33). - T13 patients (92%) achieved an AT activity at goal following supplementation. - AT activity was maintained within goal range 52% of the time during the replacement period. - Four bleeding events occurred pre-AT and 10 events post-AT administration (p = 0.26) with significantly more platelets administered post-AT (pre-AT = 0.5 units, post-AT = 4.5 units; p = 0.01). |
| Panigada 2019 | Pilot  Prospective RCT single blinded multi center controlled two arm trial | Ongoing  VV ECMO  For severe respiartory failure |  | Keep AT 80-120% vs control  Using heparin standard protocol | - Bolus of Thrombate III based on a protocol | - Ongoing |
| Iapichino 2019 | retrospective | VV ECMO  adults | 66 | AT> 70% | - Human plasma derived AT (15-30 units/kg/day) | - 34 subjects who received antithrombin concentrate, AT activity increased (from 54 ± 9 to 84 ± 13%; p < 0.001); the proportion of subjects with aPTT ratio ≥1.5 increased (from 21/34 [62%] to 31/34 [91%]; p = 0.004); heparin dosage remained constant (from 19 ± 7 to 19 ± 6 IU/kg/h; p = 0.543); and C-reactive protein decreased (from 17 ± 10 to 13 ± 9 mg/dl; p = 0.013). |
| Jones 2017 | Retrospective chart review | < 18 yo ECMO, VAD, vascular thrombosis | 12  ECMO n=2 | Serum AT levels are <80% and if the patient is requiring large doses of heparin infusion | - Thrombate or Atryn | - From time of heparin initiation to AT supplementation, patients spent a mean 4.9 ± 2.6 days of subtherapeutic infusion and required uptitration from a mean of 15.3 ± 4.4 units/kg/hr to a mean rate of 40.7 ± 9.5 units/kg/hr. - 58 % of the patients (n = 7) had a ≥10% reduction from the baseline heparin infusion rate within 48 hours of ATIII administration. - Those patients considered responders (≥10% reduction from baseline rate) had a slightly higher mean baseline AT level (76.3% ± 22.0% vs. 58.6% ± 2.7% in non-responders, p = 0.1) and were administered comparable doses of AT. - AT supplementation did appear to increase the time of therapeutic anticoagulation within the 48 hours. |
| Wong 2016 | Retrospective, multi-center, cohort | -Forty-three free-standing children's hospitals participating in Pediatric Health Information System.  -Children < 18 years of age who underwent ECMO between 2003 and 2012. | 8601 |  |  | - 1,931 of 8,601 eligible subjects (21.5%) received at least one dose of AT during their ECMO course - AT use during ECMO increased from 2.4% to 51.9% (p < 0.001) over the 10-year study period. - Subjects who received AT while on ECMO were younger (p = 0.02), had more chronic conditions (p < 0.001), and longer hospital stays (p < 0.001). - On multivariate analysis, AT use was associated with thrombotic events (odds ratio, 1.55; 95% CI, 1.36-1.77; p < 0.001), hemorrhagic events (odds ratio, 1.27; 95% CI, 1.14-1.42; p < 0.001), and longer hospital length of stays (slope coefficient, 1.05 d; 95% CI, 1.04-1.06; p < 0.001). - No difference was observed in mortality (odds ratio, 0.99; 95% CI, 0.89-1.11; p = 0.90). |
| Tzanetos 2017 [[90](#_ENREF_90)] | Retrospective | 75 VA ECMO;  2 VV ECMO  44 Neonates  Median age: 17 days | 77 | AT> 80% | - Institutional protocol - Bolus of recombinant AT (50 units/kg) followed by continuous infusion or boluses at physician discretion | - AT below 80% average of 5.2 hours per patient. - AT activity < 80% was not associated with thrombotic complications (odds ratio [OR] = 1.02, 95% confidence interval [CI] = 0.97-1.06, p = 0.86). - AT activity > 80% was not associated with bleeding complications (OR = 1.06, 95% CI = 1.01-1.11, p = 0.44). - Duration of ECMO was an independent predictor of thrombotic complications (OR = 1.08, 95% CI = 1.02-1.11, p = 0.02). - No independent predictors of bleeding complications. - AT activity correlated with anti Xa activity (r = 0.367, p < 0.001) but not with other measures of anticoagulation or heparin dose (r = 0.16, p = 0.165). - AT activity was not associated with bleeding, thrombosis; was associated with anti Xa activity, but not with traditional measures of anticoagulation. |
| Stansfield 2017 [[87](#_ENREF_87)] | Retrospective  cohort | VA ECMO > 24 hours  neonates with hypoxic respiratory failure | 162  90 standard coagulation  (control)  72 routine AT supplementation | AT>100% | - 125 IU/Kg of Thrombate III^®^ at initiation and 12 hours later (max dose 500IU) - AT level checked daily - 25 IU/Kg bolus given if AT <100% | - Infants receiving AT during ECMO had less thrombotic and similar bleeding complications compared to infants receiving standard anticoagulation therapy. - Total blood product use during ECMO was decreased (54.7±20.1 vs. 67.4±34.9mL/kg per day, p=0.001) in infants receiving AT during ECMO. - Tighter control of ACT and higher anti-Xa levels were observed in the AT cohort during first days of ECMO support. - ECMO circuit lifespan did not differ between groups. |
| Nelson 2017 [[63](#_ENREF_63)] | Retrospective case-control | VA ECMO | 14 patients -  continuous infusion AT through a standardized replacement protocol  Historic controls receiving intermittent AT doses | 80-100% | - Continuous infusion (ATryn^®^) using a standardized replacement protocol vs. historic controls (Thrombate III^®^) receiving intermittent AT doses at physician discretion | - Patients receiving CI AT protocol spent more time in goal ACT range (71.9% vs 52.2%, p < 0.0001). - Mean daily AT activity increased in study group (77.3% versus 68.6%, p = 0.04). - No statistical differences in number of heparin dose changes per day (3 versus 3.22, p = 0.90) were noted. - Only 28% of historic controls (intermittent AT) achieved normal AT activity vs 80% of study patients (p = 0.24). - Maximum heparin dose was lower in CI protocol group (p < 0.01). - CI of AT demonstrated increased time within goal ACT at a lower heparin dose, no increase in hemostatic complications, trend toward fewer heparin changes, lower blood product usage. |
| Martucci 2017 [[91](#_ENREF_91)] | Observational | VV ECMO  Adult | 82 | >100% | - AT level checked daily and replaced as needed to keep AT >100% | - Inverse relationship with transfusion volume and mortality |
| Wong 2016 | Retrospective, multi-center, cohort | Forty-three free-standing children's hospitals participating in Pediatric Health Information System.  Children < 18 years of age who underwent  ECMO  between 2003 and 2012. | 8601 |  |  | - 1,931 of 8,601 eligible subjects (21.5%) received at least one dose of AT during their ECMO course - AT use during ECMO increased from 2.4% to 51.9% (p < 0.001) over the 10-year study period. - Subjects who received AT while on ECMO were younger (p = 0.02), had more chronic conditions (p < 0.001), and longer hospital stays (p < 0.001). - On multivariate analysis, AT use was associated with thrombotic events (odds ratio, 1.55; 95% CI, 1.36-1.77; p < 0.001), hemorrhagic events (odds ratio, 1.27; 95% CI, 1.14-1.42; p < 0.001), and longer hospital length of stays (slope coefficient, 1.05 d; 95% CI, 1.04-1.06; p < 0.001). - No difference was observed in mortality (odds ratio, 0.99; 95% CI, 0.89-1.11; p = 0.90). |
| Lavandosky 2015 | Case report | VV ECMO  ADULT 39 YO | 1 | AT> 70%  Protocol for supplementation based on body weight and AT level | ATryn | - ATryn successfully restored AT activity to >70% in an adult receiving VV- ECMO support. - AT levels were normalized over a relatively short period of time. |
| Wong 2015 [[85](#_ENREF_85)] | Retrospective observational | VA ECMO for respiratory failure  pediatric population; Neonates and children | 64;  34 controls  30 AT treated | AT >80% | Thrombate III^®^ bolus if AT level less than 80%:  1) at physician discretion  2) if UFH requirement more than 40 U/kg/h or 3) if presence of an extracorporeal thrombosis | - 77 AT doses given (1-8 doses/patient) - Intermittent, on-demand dosing of AT concentrate increased AT levels, but not typically to the targeted level. - Patients who received AT concentrate also had decreased heparin requirements at least 12 hours after dosing. - Increased circuit failure in AT treated group - No differences were noted in the measured clinical endpoints. |
| O’meara 2015 | Retrospective case series | Pediatric Cardiac ICU | 22 | Heparin protocol  AT< 100% and heparin infusion rate > 45 units/kg/hr or AT < 50% independent of heparin dose | 500 IU  Thrombate III | - Patients received mean 4±3 doses of AT per ECMO run, and 1.2±1.5 doses per 24 hours. Heparin dose was 46 unit/kg/hr before AT and 37 unit/kg/hr after, p < 0.001; anti-Xa rose from 0.36 to 0.45 IU/ml after AT, p < 0.001. PTT changed from 114 to 103 seconds, p = 0.08. - AT levels increased from 65% to 110% 8 hours after AT treatment, p < 0.001. |
| Wong 2015 [[85](#_ENREF_85)] | Retrospective observational | VA ECMO for respiratory failure  pediatric population; Neonates and children | 64;  34 controls  30 AT treated | AT >80% | Thrombate III^®^ bolus if AT level less than 80%:  1) at physician discretion  2) if UFH requirement more than 40 U/kg/h or  3) if presence of an extracorporeal thrombosis | - 77 AT doses given (1-8 doses/patient) - Intermittent, on-demand dosing of AT concentrate increased AT levels, but not typically to the targeted level. - Patients who received AT concentrate also had decreased heparin requirements at least 12 hours after dosing. - Increased circuit failure in AT treated group - No differences were noted in the measured clinical endpoints. |
| Northtrop 2015 [[66](#_ENREF_66)] | Retrospective | VA ECMO | 366 ECMO runs;  261 (old protocol)  105 (new protocol) | AT>100% | - ECMO anticoagulation lab protocol (anti-Xa, TEG, AT) - Recombinant AT was dosed if level was low for age and the heparin > 60 U/kg/hr. - AT checked 2 hours after of bolus | - Median blood product usage for packed RBCs, FFP, platelets, and cryoprecipitate decreased significantly after protocol initiation. - The occurrence of cannula site bleeding decreased from 22% to 12% (*p* = 0.04), and surgical site bleeding decreased from 38% to 25% (*p* = 0.02). - Median ECMO circuit life increased from 3.6 to 4.3 days (*p* = 0.02). - A trend toward increased patient survival was noted, but did not reach statistical significance. |
| Ryerson 2014 [[82](#_ENREF_82)] | Retrospective | VA ECMO  Median age 2.9 months  (IQR 0.6-12.6) | 36 | >50% | - 1000 Units of AT concentrate (lyophilized reconstituted) - Dose is given if AT < 50% and other markers are present (increasing heparin dose, subtherapeutic anti Xa and worsening circuit condition) | - Mean dose of AT given 241 U/kg (199-283) - 78 doses given during study period - Mean AT level pre- and post-administration was 0.40 and 0.93 U/ml, respectively - Mean anti-Xa level pre- and post-AT administration was 0.23 and 0.41 U/ml, respectively - The administration of high dose - AT decreases UFH dose requirements - There were no adverse bleeding and thrombotic or allergic events within 1 week of AT administration |
| Byrnes 2014 [[84](#_ENREF_84)] | Retrospective cohort | VA ECMO > 72 hr  Median age 227 days in those supplemented vs. 455 days in those non supplemented  45 ECMO runs | 40 | AT>70% | - Bolus of Thrombate III^®^ at physician discretion | - No difference in heparin infusion rate (*p* = 0.245) as a result of AT Tx. - Anti-Xa levels were lower before AT administration (*p* < 0.001) and were increased after AT administration (*p* < 0.001) - There was an increased frequency of circuit failure in AT treatment group compared with nontreatment group (*p* = 0.018). - Neither heparin responsiveness nor circuit life was enhanced by daily AT Tx for activity <70% |
| Niimi 2014 [[55](#_ENREF_55)] | Retrospective | VA ECMO  median age: 1 month (range, 1 day to 3.75 years). | 4 case reports | >80% | - ATryn^®^, FFP or AT< 80% or heparin dose >40 units/kg/hr with little to no extension of ACT | - The median AT dose: 368 IU/kg/day (range, 104-520 IU/kg/day) to obtain AT activity level of 80-120%. - Average time to reach AT target:12.7 hours (range, 11-17 hours) - More frequent AT checking is needed |
| Perry 2013 [[86](#_ENREF_86)] | Retrospective case control | Patients with and without Congenital Diaphragmatic Hernia (CDH)  Age < 4 weeks old | Treated before (37) and after (38) an AT protocol was implemented  CDH pre AT group n=11  CDH post AT group n=12  Non CDH pre AT group n=26  Non CDH post AT group n=26 | AT>65% | - One vial (500-600 IU) of Thrombate III^®^ at cannulation - AT level measured daily - AT replacement if AT < 65% with 1 vial of Thrombate III^®^ | - Implementation of a strict guideline for AT administration in neonates is associated with a decreased need for blood products transfusions during the first 3 days of ECMO: - Patients with CDH received less fresh frozen plasma (FFP) and platelets after AT administration was introduced (78.1 ± 19.2 ml/kg vs. 27.8 ± 6.2 ml/kg, p<0.007 and 67.8 ± 8.6 ml/kg vs. 47.8 ± 8.4 ml/kg, p=0.05 respectively); - FFP and platelet administration in patients without CDH was not different between the two periods. - Patients with and without CDH received less packed red blood cell transfusions after AT protocol was introduced (230 ± 51.5 ml/kg vs. 73.8 ± 9.7 ml/kg, p<0.002 and 173.2 ± 22.2 ml/kg vs. 66.0 ± 6.6 ml/kg, p<0.001, respectively). - Cryoprecipitate was not different in patients with and without CDH between the two periods (13 ± 2.9 ml/kg vs. 15.9 ± 7.2 ml/kg, p=NS and 6.1 ± 1.8 ml/kg vs. 3.4 ± 0.6 ml/kg, p=NS, respectively). |
| Niebler 2011 [[8](#_ENREF_8)] | Retrospective cohort | VA ECMO  Median age: 0.3 years (range 1 day-19.5 years). | 28 | AT>80% | - Thrombate III^®^ given as a bolus at physician discretion - Dose determined by physician and rounded up for waste control | - AT activity level increased significantly at 8 and 24 h after administration without a significant effect on heparin drip rate, ACT, measures of bleeding or pRBC transfusion volume when titrated using ACT - Paucity of bleeding complications when AT was supplemented |
| Agati 2006 [[83](#_ENREF_83)] | Observational | VA ECMO for post cardiotomy cardiorespiratory failure  Pediatric Population  Protocol based that was modified during study period | 11 | >100% | - Continuous IV infusion of AT with intermittent heparin - AT levels checked every 4 hours - Heparin infusion rate 10-20U/kg/h to achieve ACT 180-200 seconds | - Decreased incidence of surgical revision for bleeding in the first 48 hours |
| Urlesberger 1996 [[89](#_ENREF_89)] | Prospective cohort | Term neonates  VA ECMO | 7 |  | - Mean dose 73 IU/Kg of AT over 24 hour continuous infusion - Anti Xa titrated to 035-0.7 U/mL | - AT levels increased while markers of coagulation, prothrombin fragment 1.2 and thrombin-AT complexes, decreased over time, suggesting improved heparin effect |
| VA, Venous Arterial; VV, Venous Venous; ECMO, Extracorporeal Membrane Oxygenation; AT, antithrombin; pRBC, packed red blood cells; FFP, Fresh Frozen Plasma; AT, Antithrombin; IU, international unit; hr, hour | | | | | | |
